# Protocol for a Multicenter, Prospective, Observational Pilot Study on the Implementation of Resource-Stratified Algorithms for the Treatment of Severe Traumatic Brain Injury Across Four Treatment Phases: Prehospital, Emergency Department, Neurosurgery, and Intensive Care Unit

**DOI:** 10.1227/neu.0000000000002004

**Published:** 2022-05-03

**Authors:** Dylan P. Griswold, Nancy Carney, Nicolas M. Ballarini, Laura L. Fernandez, Angelos Kolias, Peter J. Hutchinson, Andres M. Rubiano

**Affiliations:** * Division of Neurosurgery, Department of Clinical Neurosciences, University of Cambridge, Cambridge, UK;; ‡ NIHR Global Health Research Group on Neurotrauma, University of Cambridge, Cambridge, UK;; § School of Medicine, Stanford School of Medicine, Stanford, California, USA;; ‖ School of Medicine, Oregon Health and Science University, Portland, Oregon, USA;; ¶ MEDITECH Foundation, Cali, Colombia;; # Department of Neurosurgery, El Bosque University, Bogota, Colombia;; ** Department of Neurosurgery, Valle Salud Clinics, Cali, Colombia

**Keywords:** Resource-stratified guidelines, TBI, Traumatic brain injury, Protocol, Neurotrauma, LMICs

## Abstract

Severe traumatic brain injury (sTBI) is a public health issue with great disparity among low- and middle-income countries where the implementation of evidence-based guidelines is challenging because resources are often unavailable. A consensus process including experts in the prehospital, emergency department, neurosurgery, and intensive care unit took place in Colombia to develop a set of stratified protocols called BOOTStraP, targeting resource-poor environments, but it has not been systematically implemented and tested.

**OBJECTIVE:**

To identify the facilitators of, and barriers to, collecting data about patients with sTBI and to implement a stratified protocol across the treatment phases of prehospital, emergency department, neurosurgery, and intensive care unit in low-resource settings. We also aim to identify a possible association between adherence to these protocols and outcomes for these patients.

**METHODS:**

A prospective, observational, before and after, pilot study will be performed in three phases as follows: before implementation, implementation, and after implementation. The BOOTStraP protocols will be implemented in three Colombian centers.

**EXPECTED OUTCOMES:**

We expect to find numerous barriers during the implementation phase. We also expect moderate adherence to the protocols. However, we expect to find an increase in the survival rate to hospital discharge and an improvement in neurological outcomes at discharge.

**DISCUSSION:**

This pilot study will serve as a first step to identify variables that are critical to successful implementation, to be considered for the design of a future large-scale international study to measure the effectiveness of resource-based protocols and to improve outcomes from sTBI.

**ABBREVIATIONS:**
DC: data collector
ED: emergency department
EMS: Emergency Medical Service
GOS-E: Glasgow Outcome Scale Extended
LMICs: low- and-middle income countries
RTAs: road traffic accidents
SC: study coordinator
sTBI: severe traumatic brain injury.

## GENERAL INFORMATION

### Protocol Title

Beyond One Option for the Treatment of Severe Traumatic Brain Injury: BOOTStraP.

### Protocol Registration Number

U1111-1272-0282 version 1.

### Name of Funder

National Institute for Health Research Biomedical Research Centre.

### Address of Funder

University College London Hospitals, Suite A, 1st Floor, Maple House, 149 Tottenhan Court Road, London W1T 7DN, UK.

### Name and Title of Investigators

Prof Andres M. Rubiano, MD, PhD(c)

Prof Peter J. A. Hutchinson, MBBs, PhD, FRCS

Dr Angelos Kolias, MD, PhD, FRCS

Dr Nancy Carney, PhD

Dylan Griswold, BA, MD(c), PhD(c)

Dr Laura Fernandez, MD

Nicolas M. Ballarini, PhD

### Name and Addresses of Medical Centers

Research site 1: Clinica Uros Neiva, Cra. 6 #16-35, Neiva, Huila, Colombia.

Research site 2: Clinica La Sagrada Familia, Cr. 15, Cl. 10 Esquina, Armenia, Quindio, Colombia.

Research site 3: Clinica Putumayo, Cr. 48 #10-29 862060Puerto Asis, Putumayo, Colombia.

## RATIONALE AND BACKGROUND INFORMATION

Severe traumatic brain injury (sTBI) is a public health crisis that disproportionately affects low- and-middle income countries (LMICs), where suffering an sTBI more than doubles the risk of death^1^ and where more than 90% of trauma-related deaths occur.^2^ Road traffic accidents (RTAs) are the most frequent cause of sTBI, especially among the economically productive population, costing at least 3% of their gross domestic product each year.^3,4^ LMICs are particularly vulnerable to economic loss, given that 93% of all road traffic deaths occur in LMICs, despite owning less than 60% of the world's vehicles.^4^

Colombia is one of 68 countries where RTA deaths have increased since 2010.^4-6^ Of the nearly 7000 deaths due to RTAs that occurred in Colombia in 2019, more than 3600 deaths were motorcycle operators. In countries such as Colombia with a high incidence of sTBI, evidence-based guidelines are essential for decreasing risk of mortality and serious morbidity.

Although evidence-based guidelines for the management of sTBI exist, their applicability in low-resource environments is limited because the resources required to follow the guidelines are often unavailable. To address this gap, a consensus process involving clinical experts was conducted in Colombia to develop a series of management protocols to articulate treatment options for TBI specific to different levels of resources and system complexity across the prehospital, emergency department (ED), neurosurgery, and intensive care unit (ICU) phases. The set of protocols is called BOOTStraP (Beyond One Option for Treatment of Traumatic Brain Injury: A Stratified Protocol).

The BOOTStraP protocols are divided into 4 main categories, which are based on 13 recommendations from 20 clinical experts as follows: (1) management in a basic vs advanced ambulance (prehospital setting), (2) ED care in a low vs medium-high complexity institution with or without access to a CT scan, (3) neurosurgery department, and (4) intermediate or ICU availability. Each of them is subcategorized according to the TBI severity and can be used as a guiding tool by different levels of health professionals (eg, Emergency Medical Service [EMS] technician, nurses, physicians, etc.)

The user can select from a menu of treatment options depending on what resources are available (medicine, equipment, clinician training, and skill) to follow best practice given the prevailing circumstances.

## STUDY GOALS AND OBJECTIVES

The 3 objectives of the study are:

1. To identify the facilitators of, and barriers to, collecting data about patients with sTBI across the treatment phases of prehospital, ED, neurosurgery, and ICU in low-resource settings.
2. To identify the facilitators of, and barriers to, implementing a stratified protocol for treatment of patients with sTBI across the treatment phases of prehospital, ED, neurosurgery, and ICU in low-resource settings.
3. To identify whether there is an association between adherence to the protocols and outcomes for patients with sTBI.

## STUDY DESIGN

This is a prospective, observational, before and after, pilot investigation of treatment process and outcomes for patients with sTBI, who are transported by any means to a medical center for treatment. Data will be collected for each patient through the four treatment phases of prehospital, ED, neurosurgery, and ICU or until the patient is dead. Data will be abstracted from hospital medical records; requirement for consent has been waived by the ethics committee. We will collect data on 48 patients in each of the before and after phases of the study.

### Inclusion Criteria

- Patients age ≥15 years
- Clinical indications of sTBI
- Glasgow Coma Scale ≤8 on hospital arrival, or within 48 hours of admission, or Glasgow Coma Scale ≥9 on hospital arrival, but who are either admitted to the ICU or undergo surgery within 48 hours of admission.

### Exclusion Criterion

- Patients dead at the scene on EMS arrival (patients who die during transport, either by EMS or private means) are included).

### Sample Size

- Forty-eight patients in both before and after phase (N = 96).

From initiation of the “before” phase, data for each patient will be collected from initial patient encounter to hospital discharge, until sample size is met. The BOOTStraP protocol will be implemented during the month following the end of initial data collection. After implementation (“after” phase), data for each patient will be collected from initial patient encounter to hospital discharge, until sample size is met.

## METHODOLOGY

Study timeline is described in Figure. Study month 1 was initiated in September 2021.

**FIGURE.**
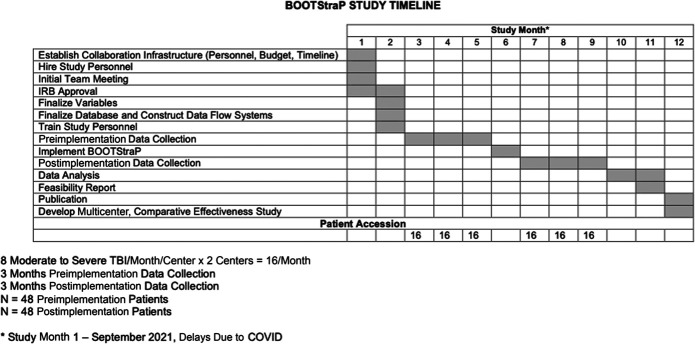
BOOTStraP study timeline.

The treatment protocols to be implemented in the three centers are provided in **Supplement 1**, http://links.lww.com/NEU/D140. Variables to be collected across the four phases are provided in the case report forms (CRFs) in **Supplement 2**, http://links.lww.com/NEU/D141.

### Categories of Variables

- Phenomenological variables (narratives)
- Adherence (derived from objective measures)
- Patient outcomes
  Survival to hospital discharge
  Glasgow Outcome Scale Extended (GOS-E) at discharge.

### Data Collection

- Each day during active data collection, the data collector (DC) for each site accesses the ED records and identifies patients admitted during the previous 24 hours who may meet inclusion criteria for the study.
- The DC ascertains information about the potential included patients from ED records and attending caregivers and identifies those screened and excluded and those screened and included.
- Data are transcribed from ED records and caregiver notes directly into a web-based database.
- The DC completes data abstraction for included patients.
- The DC identifies the location and status of newly included patients and transcribes data about each patient into the database, up to and including the current status.
- After completing data abstraction for new patients entered within the previous 24 hours, the DC identifies the location and status of patients previously included into the study and transcribes data about each patient into the database, up to and including the current status.
- Each day during active data collection, the study coordinator (SC) accesses the electronic records and reviews new data entries for errors and missing data.
- Twice monthly, the SC compares abstracted data with chart records and meets with the site DC to discuss errors and missing data.
- The SC and DC make any revisions together, directly into the database.
- During these visits, the SC records narrative information about reasons for errors and missing data, what is working and not working, and the DCs' perspectives on the barriers to and facilitators of conducting the study.
- The SC transcribes this narrative information into a qualitative data collection/analysis database.

## DISCUSSION

This is the first study to develop, implement, and test resource-stratified protocols for neurotrauma in low-resource settings. It is an important first step toward conducting a much larger multicenter, international study. A feasibility study asks whether something can be done, should we proceed with it, and if so, how? By evaluating the barriers and facilitators to implementing the protocols in these three centers in Colombia, we will better understand the feasibility of implementing the protocols in a larger, international trial. Ultimately, this study will generate data that will serve as an important evidence base from which comprehensive guidelines for treatment of sTBI in LMICs can be developed.

## TRIAL STATUS

The current status of the trial is in the preimplementation stage.

## SAFETY CONSIDERATIONS

Risks to the patients are proportional to the level of experience of the providers. These protocols are based on clinical consensus and have not yet been tested for efficacy and associated mortality and morbidity.

## FOLLOW-UP

Patients will be followed to discharge.

## DATA MANAGEMENT AND STATISTICAL ANALYSIS

The data will be recorded in a web database and handled and stored to enable accurate reporting, analysis, and interpretation. The statistician will also periodically review the data entered for completeness and accuracy and raise any queries in the system if necessary. Changes in the data entered will be recorded in an audit trail including initials, dates, and the reason for change. The database will be locked for analysis once all necessary actions have been taken to ensure the data are complete and accurate.

Demographic and baseline data will be summarized descriptively for all participants by phase (before and after). Categorical data will be summarized as frequencies and percentages while continuous variables will be summarized using mean, median, minimum, maximum, and 25th and 75th percentiles.

### Phenomenological Data

We will record narrative information from personnel at each site to investigate the barriers to and facilitators of data collection and intervention implementation. These data will be entered into a qualitative data analysis instrument, and a modified Grounded Theory approach will be used to identify common themes.

### Adherence Data

Adherence will be derived by tracking if each recommended BOOTStraP resource was available (yes/no), and if yes, whether it was used (yes/no). The analysis of adherence will be presented by treatment phase (prehospital, ED, neurosurgery, and intensive care unit). The percentage of adherence to the BOOTStraP protocol will be used as a summary statistic.

### Objective Patient Status

Survival status and GOS-E at discharge will be recorded for each patient. The analysis will explore correlations between adherence levels by phase and individual treatments on outcome data. No hypothesis will be tested.

The sample size for the study was determined based on feasibility, and no formal calculation of power was performed.

### Access to Data

Access to data set will be made available on request

### Dissemination Policy

Findings of this study will be published in a peer-reviewed journal and will be presented at relevant conferences.

## QUALITY ASSURANCE

DCs will be trained in data management. The research team will perform a constant improvement and feedback system on the protocols at each of the centers during the implementation phase.

## EXPECTED OUTCOMES OF STUDY

We expect to discover (a) what level of successful implementation is possible in the context of the prevailing conditions and (b) what changes will be required to enhance that level of success. We further expect that implementation of BOOTStraP will improve survival to hospital discharge and GOS-E at discharge. We expect there will be many barriers to implementation documented, especially in the prehospital stage. We expect adherence to protocols to be modest. The overall aim of this pilot trial is to assess the feasibility of implementing these protocols in a larger multicenter international study.

## DURATION OF THE PROJECT

Before implementation data collection: 3 months.

Implementation: 2 months.

After implementation data collection: 3 months.

## PROJECT MANAGEMENT

Principal Investigators: Prof A. M. Rubiano, MD, PhD

Coinvestigators: D. P. Griswold, BA; Dr N. Carney, PhD; Dr A. K. Kolias, MD, PhD; and Prof P. J. A. Hutchinson MBBS, PhD, FRCS

Head Statistician: N. M. Ballarini, PhD

Site and Study Coordinator: L. L. Fernandez, MD

## ETHICS

This study will be performed in accordance with the Declaration of Helsinki.^7^ The BOOTStraP protocol was approved by the Institutional Review Board of both Clinica Uros and Fundacion Meditech.

## Supplemental Digital Content

**Supplement 1.** Manual of processes and procedures for BOOTStraP protocol implementation across emergency department, neurosurgery, and intensive care unit. Meditech and Clinica Uros logos used with permission from Meditech. All rights reserved. **Supplement 2.** Case report form for BOOTStraP variables.
